# Typical clinical presentation of acute myocardial infarction and confusing coronary angiography: A case report and literature review of coronary embolism

**DOI:** 10.1097/MD.0000000000033782

**Published:** 2023-05-12

**Authors:** Mo-Qing Yin, Li-Hua Fan, Yun-Hu Chen

**Affiliations:** a Department of Cardiology, Taicang TCM Hospital Affiliated to Nanjing University of Chinese Medicine, Suzhou, Jiangsu Province, China.

**Keywords:** acute coronary syndrome, aneurysm of the ventricular membranous septum, case report, coronary embolism, myocardial infarction, spontaneous coronary artery dissection

## Abstract

The purpose of this article is to discuss the characteristics, diagnosis, treatment, and outcomes of cases of multiple coronary embolism (CE) highly suspected to be caused by a dislodged aneurysm of the ventricular membranous septum (AVMS) thrombus.

**Patient concerns:**

A 35-year-old man was rushed to the Chest Pain Center of Taicang TCM Hospital Affiliated to Nanjing University of Chinese Medicine for sudden onset of chest pain. The patient had severe and persistent chest pain without relief, accompanied by sweating throughout the body.

**Diagnosis:**

An electrocardiogram showed ST-segment elevation in the inferior wall leads, and blood tests suggested elevated troponin I levels. The initial diagnosis was acute ST-segment elevation myocardial infarction. Emergency coronary angiography revealed complete occlusion of the first diagonal branch, thrombolysis in myocardial infarction grade 0 flow, and smooth remaining vessels. Complete occlusion of the left anterior descending artery unexpectedly occurred during interventional treatment. Postoperative cardiac ultrasonography revealed the presence of a thrombus within the AVMS and in the apical portion of the heart. The final diagnosis was a CE.

**Interventions:**

Intraoperatively, the diagonal branch occluded segment was dilated with a balloon and intracoronary administration of tirofiban and nitroglycerin. Postoperatively, antithrombotic therapy (aspirin, clopidogrel, and rivaroxaban) was administered.

**Outcomes:**

Ten days after admission, a repeat coronary angiography showed complete restoration of left anterior descending artery flow on its own, balloon dilation was again performed on the diagonal branch, and flow was restored to thrombolysis in myocardial infarction grade 1. Six months later, the intracardiac thrombus disappeared on repeat cardiac ultrasound.

**Lessons:**

AVMS is a potential source of embolism in patients with CE. CE has features that distinguish it from atherosclerosis, and a timely and correct diagnosis can help improve patient clinical outcomes.

## 1. Introduction

Acute coronary syndrome (ACS) is usually associated with coronary atherosclerosis; however, coronary embolism (CE) is a rare and unique cause of ACS. Approximately 3% of ACS cases and 4 to 13% of acute ST-segment elevation myocardial infarction cases are due to CE.^[1,2]^ CE is exceptionally fatal with an in-hospital mortality rate of up to 36%.^[3]^ CE is distinguished from coronary atherosclerosis in terms of treatment strategy and prognosis. The timely, accurate identification and management of CE, which involves aggressively searching for the source of the embolism, is challenging. An aneurysm of the ventricular membranous septum (AVMS) is a rare congenital malformation of the heart originating from the membranous ventricular septum. Embolic stroke has been reported to occur in 14% of patients with an AVMS,^[4]^ but no cases of an AVMS leading to CE have been reported. We report the first case of a patient with multiple CEs with acute inferior wall infarction as the first presentation. Postoperative cardiac ultrasound revealed the presence of an intra-AVMS thrombus in this patient, which was highly suspected to cause CE by thrombus dislodgement. Understanding the characteristics of CE, specific treatment, and identifying the source of embolism will help improve clinical outcomes in this patient population.

## 2. Case presentation

A 35-year-old Chinese man presented to our chest pain center on June 20, 2022, with “sudden onset of chest pain for 2 hours.” Two hours before the visit, the patient suddenly developed severe pain in the left precordial region at rest that persisted without relief and was accompanied by generalized sweating. The patient had visited other hospitals for chest pain in June 2021, at which time a cardiac ultrasound was performed suggesting the presence of an AVMS and a negative coronary computed tomography angiography. The patient did not have any other medical history and was not using medication long-term. The patient did not have a long history of heavy smoking or alcohol abuse, a history of allergies, or any family history of hereditary disease.

On physical examination, the vital signs were as follows: body temperature, 36.4°C; blood pressure, 15.2/10 kPa; heart rate, 60 beats per min; and respiratory rate, 17 breaths per min. In addition, there were no murmurs in the auscultation areas of each heart valve, no rales in the lungs, and no edema in the limbs. Neurological examination showed no positive signs.

The laboratory results were as follows: troponin I, 0.079 ng/mL (reference range: <0.023 ng/mL); D-dimer, 324 ng/mL (reference range: 80–500 ng/mL); NT-ProBNP, 306 ng/L (reference range: 300–900 ng/L); white blood cells, 10.8 × 10^9^/L (reference range: 4.5-9.5 × 10^9^/L); hemoglobin, 138 g/L (reference range: 120–165 g/L); platelets, 168 × 10^9^/L (reference range: 100–300 × 10^9^/L); blood creatinine, 68 μmol/L (reference range: 57–130 μmol/L); alanine aminotransferase, 36 U/L (reference range: 0–40 U/L); aspartate aminotransferase, 78 U/L (reference range: 0–40 U/L); and blood potassium, 4.1 mmol/L (reference range: 3.5–5.5 mmol/L).

**Figure 1.**
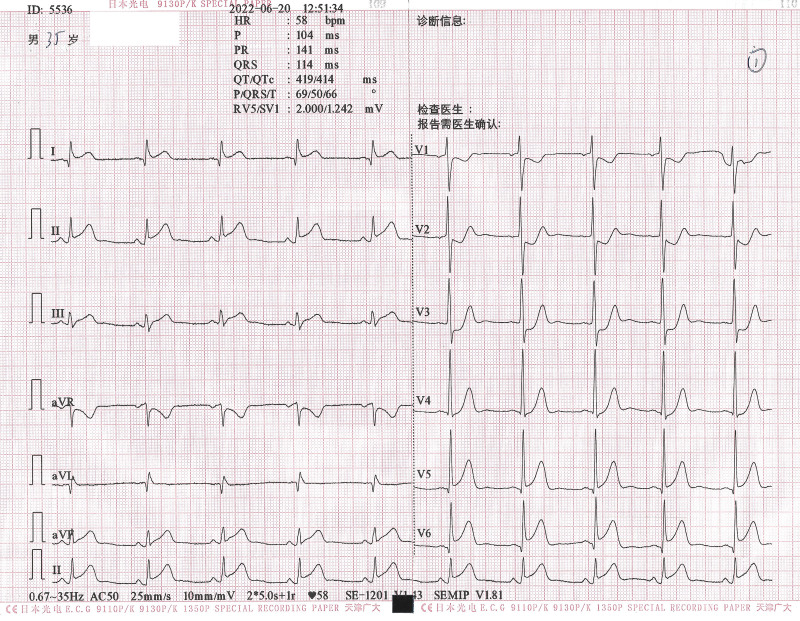
First electrocardiogram (ECG). Sinus bradycardia with a heart rate of 58 beats/min and ST-segment elevation in leads I, II, III, aVF, V5, and V6 of 0.05 to 0.2 mV.

**Figure 2.**
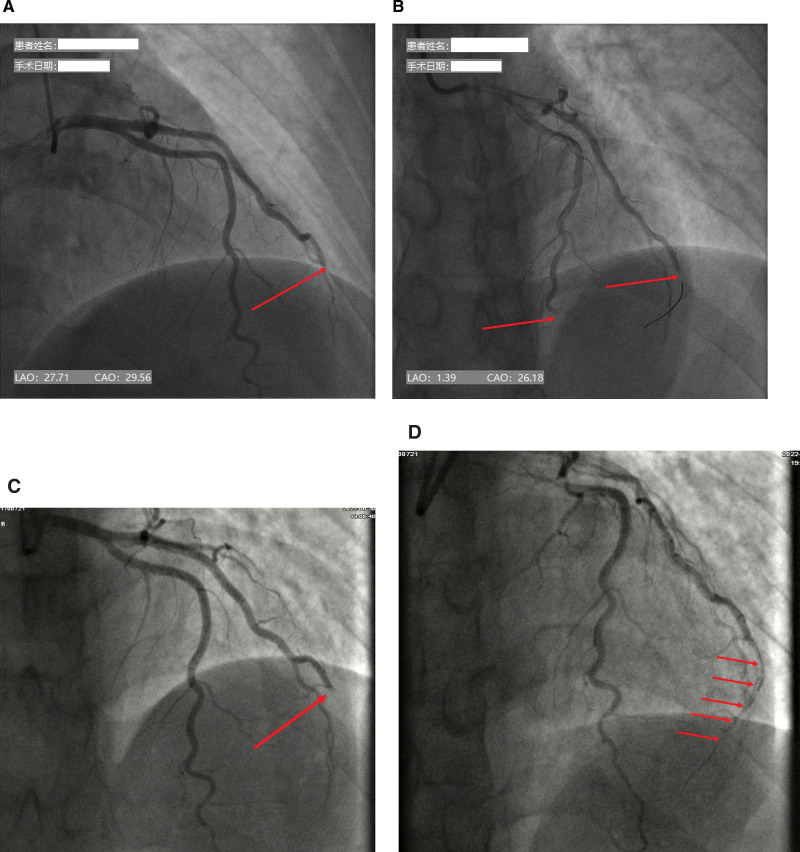
Coronary angiography (CAG) images. (A) First CAG showing diagonal branch occlusion (arrow). (B) The guidewire was delivered distally through the occluded segment of the diagonal branch, but no blood flow was seen on the repeat angiogram (right arrow). At this time, distal of the LAD was occluded (left arrow). (C) Ten days after disease onset, CAG was repeated and showed restored blood flow in the LAD, while the diagonal branch remained occluded (arrow). (D) After percutaneous transluminal coronary angioplasty (PTCA) of the diagonal branch, blood flow was restored to thrombolysis in myocardial infarction (TIMI) grade 1 flow. LAD = left anterior descending artery.

A chest computed tomography scan showed no obvious abnormality. An electrocardiogram suggested ST-segment elevation in the inferior wall leads (Fig. 1). The initial diagnosis was acute ST-segment elevation inferior wall infarction. The patient immediately received oral aspirin (0.3 g), ticagrelor (180 mg), and rosuvastatin (10 mg). Coronary angiography (CAG) was immediately performed. CAG showed that the right coronary artery, left main stem coronary artery, left anterior descending artery (LAD), and left circumflex artery were completely normal without any traces of atheromatous plaque, and the first diagonal branch was distally occluded with thrombolysis in myocardial infarction (TIMI) grade 0 flow (Fig. 2A). After unsuccessful attempts to pass the occluded segment of the diagonal branch with a Runthourth NS guidewire (Terumo Corporation in Japan, with a tip hardness of 0.8g.), the occluded segment was passed through a PILOT 50 guidewire (Abbott Laboratories in the United States, with a tip hardness of 2.0g.) supported by a microcatheter, which enabled the distal end of the guidewire to repeatedly enter the distal branch in the same direction without resistance. The occluded segment was dilated repeatedly with 1.0 mm and 1.5 mm diameter balloons, and nitroglycerin, sodium nitroprusside, and tirofiban were pushed into the coronary artery several times. After a repeat CAG, no flow was detected in the distal part of the occluded segment. We delivered the microcatheter to the distal end of the occluded segment and performed negative pressure aspiration, but no blood flow was observed. When we performed CAG again, the previously normal LAD unexpectedly developed a distal occlusion (Fig. 2B). This led us to suspect that this patient may have had a CE and not coronary atherosclerosis. We immediately inserted another Runthourth NS guidewire and delivered it with difficulty through the occluded segment of the LAD to the distal end. Eventually, the flow in the distal LAD recovered to a TIMI grade of 1, and the flow in the diagonal branch remained at a TIMI grade of 0.

**Figure 3.**
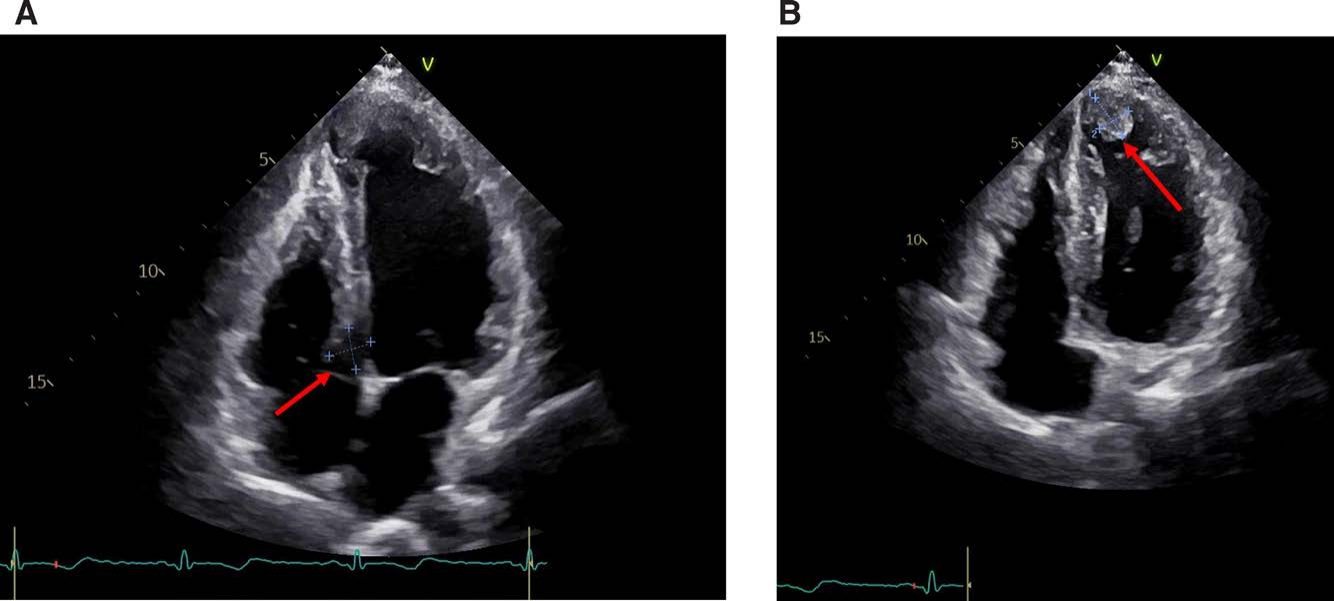
Echocardiography. (A) A membranous septal aneurysm (arrow) protruding into the right ventricular outflow tract with some hypoechoic tissue visible within it, which was considered to be a thrombus. (B) Left ventricular apical thrombus (arrow).

On the 5th postoperative day, cardiac ultrasonography revealed an AVMS with regular contour and a wide base, a sac-like structure (13 × 14 mm) protruding into the right ventricular outflow tract, with small hypoechoic sparse tissue visible inside, suggesting the possibility of thrombus (Fig. 3A). Simultaneously, the left ventricular inferior wall, posterior wall, mid-lateral wall, and entire apical region were less active, with an ejection fraction of 41%. The apical ventricular wall was relatively thin and slightly expanded outward, with multiple moderately hypoechoic attachments visible inside (the largest one was 17 mm × 12 mm) and little activity (Fig. 3B).

The patient was diagnosed with CE, an AVMS, ventricular thrombosis, and heart failure. Due to the heavy thrombus burden, rivaroxaban (10 mg/day) was added to the antiplatelet therapy (aspirin 0.1 g/day, clopidogrel 75 mg/day). On the tenth day of admission, we performed CAG again. The angiogram showed complete normalization of the LAD occlusion; however, there was still no flow in the occluded segment of the diagonal branch (Fig. 2C). The diagonal branch was again subjected to percutaneous transluminal coronary angioplasty, and a TIMI grade 1 flow was eventually restored (Fig. 2D). No episodes of atrial fibrillation were observed on 48-hour ambulatory electrocardiogram performed during hospitalization.

After 15 days of hospitalization, the patient was discharged without incident. Six months later, the patient’s repeat cardiac ultrasound showed that both the apical and intra-AVMS thrombi disappeared and that the left ventricular ejection fraction recovered to 45%. The formerly occluded segment of the coronary angiogram returned to normal. The patient refused surgical treatment for the AVMS.

## 3. Discussion

### 3.1. CE

CE is a secondary phenomenon and is usually associated with a primary disease or etiology. Common causes include atrial fibrillation, infective endocarditis, paradoxical embolism (e.g., patent foramen ovale or atrial septal defect), iatrogenic causes (e.g., surgical material, thrombus in an interventional catheter, dislodged atherosclerotic plaque), dislodged thrombus in a wall attached to the heart, autoimmune disease, or even malignancy. There may be an overlap between the various etiologies. Embolic tissue can be a thrombus, surgical material, adipose tissue, source of infection, cancerous emboli, etc.^[1,5]^ Defining the etiology and embolic tissue of CE facilitates the subsequent treatment and prognosis of the disease. However, notably, the underlying cause is unknown in some cases of CE.

CE usually appears on imaging as a truncated filling defect in 1 or more coronary arteries and lacks signs of coronary atherosclerosis. The most commonly affected vessel is the LAD, followed by the right coronary artery and left main stem coronary artery.^[5]^ Some patients present with “bystander atherosclerosis,” which makes the diagnosis of CE more challenging. During the intervention, the percutaneous transluminal coronary angioplasty guidewire encounters resistance through the embolic tissue that is usually greater than that of the lipid plaque formed in the coronary artery, making it more difficult to restore the blood flow to the distal end. Therefore, aspiration thrombectomy should also be considered. Pathological tissue examination of the aspirated material was performed to determine the nature and source of the embolic tissue. Postoperative anticoagulation therapy should be considered, and some patients with stent implantation will also need antiplatelet therapy. However, a consensus on treatment has not been reached because there is very little relevant data available.

Ventricular thrombosis is a common and severe complication of myocardial infarction and is an independent predictor of future cardiovascular events.^[6–8]^ In general, most left ventricular thrombi form within 1 to 2 weeks after an acute event.^[9]^ Epidemiological data show that 15% of patients with ST-segment elevation myocardial infarction and 25% of patients with anterior wall infarction present with left ventricular thrombus.^[10]^ Patients with left ventricular thrombosis have a higher chance of suffering complications such as bleeding, renal failure, shock, and cardiac arrest than patients without left ventricular thrombosis. However, existing studies on ventricular thrombosis are largely based on classical atherosclerosis-induced infarction, and few have examined ventricular thrombosis after CE. It has not been reported whether the incidence of ventricular thrombosis after CE is different from that of atherosclerosis-induced ventricular thrombosis.

Patients with CE usually have a high probability of developing other systemic embolisms, and a retrospective analysis showed that up to 15% of patients with CE had clinical evidence of stroke.^[11]^ Therefore, to reduce the risk of a recurrent embolic event, patients should be screened for the source of embolic material and systemic embolism after surgery. The prognosis of acute myocardial infarction secondary to CE may be worse than that of myocardial infarction secondary to coronary atherosclerosis, possibly because sudden CE does not allow sufficient time for collateral circulation to develop. In addition, the prognosis of CE is significantly correlated with primary disease.

### 3.2. Differentiation from spontaneous coronary artery dissection (SCAD)

SCAD is another rare cause of ACS and has a similar angiographic presentation to CE; however, the 2 are distinct conditions that require differentiation. SCAD occurs frequently in women under 50 years of age and is characterized by true and false lumens caused by hematomas within the coronary mesothelium, most often in the LAD.^[12,13]^ The disease is generally observed to heal spontaneously, so conservative treatment is preferred. The etiology may be related to fibromuscular dysplasia, pregnancy, autoimmune diseases, or mental stress.^[13]^ CAG remains an important diagnostic modality for SCAD. However, CE can mimic the angiographic features of SCAD, making the differential diagnosis challenging. Coexisting fibromuscular dysplasia, hereditary connective tissue disease, pregnancy-related disease, and complete coronary recovery support the diagnosis of SCAD.^[14,15]^ SCAD is uncommon in cases of non-acute onset ACS, in male patients, and in young (<25 years) or very old (>80 years) individuals. CE usually has a primary event leading to an embolic event, enabling the source of the embolism to be identified. Based on angiographic images, Yip and Saw classify SCAD into 3 types^[16]^; double-lumen and long and smooth stenosis are included in the classification criteria, but these criteria do not include complete occlusion of the vessel due to SCAD. Therefore, vascular occlusions not meeting the criteria of types 1 to 3 may be classified as type 4. Both CE and SCAD (type 4) present with occlusion of the coronary arteries, whereas the rest of the vessels are essentially normal; however, truncated occlusion of multiple coronary branches is highly suggestive of CE. SCAD (type 4) is usually characterized by progressive thinning of the upstream coronary artery before the occluded segment and the presence of a double-lumen appearance in the long segment of the stenosis, a feature that can be explained by the pathological features of SCAD. SCAD (type 4) rarely presents with truncation of the coronary artery, as single-vessel changes are more common. Although the above features provide a diagnostic and differential diagnosis in most patients, there remains uncertainty in using CAG alone to distinguish SCAD (type 4) from CE. Furthermore, the use of CAG alone can leave diagnostic doubt, and intracoronary imaging is often helpful. Some patients require follow-up with CAG or coronary computed tomography angiography. Most patients with SCAD heal after 1 month, and while some patients with CE due to thrombosis may heal, the healing time is usually shorter than that in SCAD patients.

### 3.3. AVMS

An AVMS is a rare congenital malformation of the heart. Despite current advances in ultrasound and magnetic resonance diagnostic techniques, it is still difficult to determine the prevalence of this condition. An AVMS occurs below the septal leaflets of the tricuspid valve, and the absence of localized septal myocardium results in an aneurysm-like change that anatomically manifests as a fibrous wall sac protruding from the septal membrane into the right ventricular outflow tract.^[17,18]^ The diagnosis is usually confirmed by echocardiography, cardiac magnetic resonance imaging, or computed tomography. In most cases, the AVMS itself is not hemodynamically significant, but the presence of local hemodynamic alterations and local ventricular wall motion abnormalities within this particular cystic structure leads to the risk of potential complications, such as rupture, thrombosis, aortic valve prolapse, tricuspid valve insufficiency, right ventricular outflow tract obstruction, endocarditis, and arrhythmias.^[19,20]^ The presence of turbulent flow within the AVMS, which is prone to thrombus formation, promotes the development of embolic events. Some reports suggest that intra-aneurysmal thrombosis is present in 11.6% of adult patients with AVMS,^[4]^ which is a clear independent predictor of embolic stroke. CE events due to an AVMS are rarely reported because the coronary arteries do not follow a relatively linear trajectory of the left ventricular outflow tract. After the thrombus dislodges from the left ventricle, it has a greater chance of entering the carotid artery or distal body circulation with high-velocity blood flow; therefore, there is a natural anatomical mechanism that protects against thrombotic events in the coronary arteries. In adults, if an AVMS does not spontaneously subside or shrink, surgical intervention is less likely to be required if there are no significant complications, and regular cardiac ultrasound is performed instead. Surgical repair is recommended if there are significant complications such as cerebral embolism and hemodynamic abnormalities. In the current case study, a mass found within the AVMS was suggestive of thrombosis, and anticoagulation therapy was reasonable.

## 4. Conclusion

AVMS is a potential source of embolism in patients with CE. The risk of thrombotic events in patients with an AVMS is higher than previously reported. CE has features that distinguish it from atherosclerosis and SCAD, and a timely and correct diagnosis can help improve patient clinical outcomes.

## Acknowledgments

Special thanks to Professor Shou-Peng Chen of Nanjing University of Chinese Medicine for providing data retrieval services for this work.

## Author contributions

**Data curation:** Mo-Qing Yin, Li-Hua Fan.

**Investigation:** Mo-Qing Yin.**Methodology:** Yun-Hu Chen.

**Writing – original draft:** Mo-Qing Yin, Li-Hua Fan.

**Writing – review & editing:** Yun-Hu Chen.

## References

[1] Raphael CE Heit JA Reeder GS. Coronary embolus: an underappreciated cause of acute coronary syndromes. JACC Cardiovascular Intervent. 2018;11:172–80. 10.1016/j.jcin.2017.08.057 29348012

[2] Popovic B Agrinier N Bouchahda N. Coronary embolism among ST-segment-elevation myocardial infarction patients: mechanisms and management. Circ Cardiovascular Intervent. 2018;11:e005587. 10.1161/CIRCINTERVENTIONS.117.005587 29311288

[3] Vendittelli PS Botros B Rosman HS. Coronary artery embolism: two case reports and a review of the literature. Am J Med Sci. 2019;357:333–7. 30545698 10.1016/j.amjms.2018.11.003

[4] You JY Chun EJ Choi SI. Clinical significance of incidentally detected aneurysms of the membranous ventricular septum in adults by multidetector computed tomography. Am J Cardiol. 2015;115:354–9. 25491239 10.1016/j.amjcard.2014.11.010

[5] Lacey MJ Raza S Rehman H. Coronary embolism: a systematic review. Cardio Revascularization Med. 2020;21:367–74. 10.1016/j.carrev.2019.05.012 31178350

[6] Cruz Rodriguez JB Okajima K Greenberg BH. Management of left ventricular thrombus: a narrative review. Annal Trans Med. 2021;9:520. 10.21037/atm-20-7839 PMC8039643 33850917

[7] Katsouras CS. Left ventricular mural thrombus. J Am Coll Cardiol. 2020;76:484. 10.1016/j.jacc.2020.05.067 32703521

[8] Lattuca B Bouziri N Kerneis M. Antithrombotic therapy for patients with left ventricular mural thrombus. J Am Coll Cardiol. 2020;75:1676–85. 32273033 10.1016/j.jacc.2020.01.057

[9] Albaeni A Chatila K Beydoun HA. In-hospital left ventricular thrombus following ST-elevation myocardial infarction. Int J Cardiol. 2020;299:1–6. 31371119 10.1016/j.ijcard.2019.07.070 PMC6891157

[10] McCarthy CP Vaduganathan M McCarthy KJ. Left ventricular thrombus after acute myocardial infarction: screening, prevention, and treatment. JAMA Cardiol. 2018;3:642–9. 29800958 10.1001/jamacardio.2018.1086

[11] Shibata T Kawakami S Noguchi T. Prevalence, clinical features, and prognosis of acute myocardial infarction attributable to coronary artery embolism. Circulation. 2015;132:241–50. 26216084 10.1161/CIRCULATIONAHA.114.015134

[12] Meng PN Xu C You W. Spontaneous coronary artery dissection as a cause of acute myocardial infarction in young female population: a single-center study. Chin Med J (Engl). 2017;130:1534–9. 28639567 10.4103/0366-6999.208245 PMC5494915

[13] Adlam D Tweet MS Gulati R. Spontaneous coronary artery dissection: pitfalls of angiographic diagnosis and an approach to ambiguous cases. JACC Cardiovascular Intervent. 2021;14:1743–56. 10.1016/j.jcin.2021.06.027 PMC8383825 34412792

[14] Hayes SN Tweet MS Adlam D. Spontaneous coronary artery dissection: JACC state-of-the-art review. J Am Coll Cardiol. 2020;76:961–84. 32819471 10.1016/j.jacc.2020.05.084

[15] Iismaa SE Hesselson S McGrath-Cadell L. Spontaneous coronary artery dissection and fibromuscular dysplasia: vasculopathies with a predilection for women. Heart Lung Circulation. 2021;30:27–35. 32713767 10.1016/j.hlc.2020.05.110 PMC7710561

[16] Yip A Saw J. Spontaneous coronary artery dissection-a review. Cardiovascular Diagnosis Ther. 2015;5:37–48. 10.3978/j.issn.2223-3652.2015.01.08 PMC4329168 25774346

[17] De Almeida MC Sanchez-Quintana D Anderson RH. The membranous septum revisited: a glimpse of our anatomical past. Clin Anatomy (New York, NY). 2021;34:178–86. 10.1002/ca.23599 32249445

[18] Abdul Jabbar A Mufti O Mazur W. Isolated aneurysms of the membranous ventricular septum without residual shunts: systematic review and description of 3 cases in adults. J Ultrasound Med. 2017;36:869–78. 28230264 10.7863/ultra.16.02087

[19] Di Cesare E Di Sibio A Lanni G. Magnetic resonance imaging of AMS (aneurysm of the membranous septum), review of the literature and case report. J Radiol Case Rep. 2014;8:9–15. 10.3941/jrcr.v8i5.1715 PMC4242064 25426225

[20] Carcano C Kanne JP Kirsch J. Interventricular membranous septal aneurysm: CT and MR manifestations. Insights Imag. 2016;7:111–7. 10.1007/s13244-015-0456-3 PMC4729708 26687514

